# Wet-Chemical Synthesis of TiO_2_/PVDF Membrane for Energy Applications

**DOI:** 10.3390/molecules28010285

**Published:** 2022-12-29

**Authors:** Muhammad Saleem, Munirah D. Albaqami, Aboud Ahmed Awadh Bahajjaj, Fahim Ahmed, ElSayed Din, Waqas Ul Arifeen, Shafaqat Ali

**Affiliations:** 1 Department of Physics, The Islamia University of Bahawalpur, Bahawalpur 63100, Pakistan; 2 Department of Chemistry, College of Science, King Saud University, Riyadh 11451, Saudi Arabia; 3 Department of Physics, Division of Science and Technology, University of Education, Lahore 54000, Pakistan; 4 Faculty of Engineering and Technology, Future University in Egypt, New Cairo 11835, Egypt; 5 School of Mechanical Engineering, Yeungnam University, Gyeongsan-si 38541, Gyeongsangbuk-do, Republic of Korea; 6 Department of Environmental Sciences, Government College University, Faisalabad 38000, Pakistan; 7 Department of Biological Sciences and Technology, China Medical University, Taichung 40402, Taiwan

**Keywords:** TiO_2_ nanoplates, PVDF, wet-chemical, negative electrode, energy storage

## Abstract

To satisfy the ever-increasing energy demands, it is of the utmost importance to develop electrochemical materials capable of producing and storing energy in a highly efficient manner. Titanium dioxide (TiO_2_) has recently emerged as a promising choice in this field due to its non-toxicity, low cost, and eco-friendliness, in addition to its porosity, large surface area, good mechanical strength, and remarkable transport properties. Here, we present titanium dioxide nanoplates/polyvinylidene fluoride (TiO_2_/PVDF) membranes prepared by a straightforward hydrothermal strategy and vacuum filtration process. The as-synthesized TiO_2_/PVDF membrane was applied for energy storage applications. The fabricated TiO_2_/PVDF membrane served as the negative electrode for supercapacitors (SCs). The electrochemical properties of a TiO_2_/PVDF membrane were explored in an aqueous 6 M KOH electrolyte that exhibited good energy storage performance. Precisely, the TiO_2_/PVDF membrane delivered a high specific capacitance of 283.74 F/g at 1 A/g and maintained capacitance retention of 91% after 8000 cycles. Thanks to the synergistic effect of TiO_2_ and PVDF, the TiO_2_/PVDF membrane provided superior electrochemical performance as an electrode for a supercapacitor. These superior properties will likely be used in next-generation energy storage technologies.

## 1. Introduction

The constant consumption of conventional fossil fuels and the resulting increase in environmental problems have made the development of low-carbon emissions and environmentally friendly methods of energy storage an urgent necessity [1,2]. Supercapacitors (SCs) have led to their widespread consideration as renewable sources due to the favorable electrochemical performance of rapid charging/discharging methods, high power density, and extended cycle stability [3,4]. Depending on their charge storage mechanism, SCs are classified as electric double-layer capacitors (EDLCs) or pseudocapacitors (PCs) [5]. Compared to EDLCs, PCs can offer diverse redox states and achieve high specific capacitance because of the reversible redox reactions at the electrode/electrolyte interface and on the active materials’ surfaces [6].

Electrode materials can significantly affect the use of such energy storage devices for practical applications [7,8]. Widespread functional materials, including activated carbon [9], carbon nanotubes [10], graphene [11], and transition metal oxides [12,13] have significant potential because of their stability and remarkable electrochemical performance. However, most commercial carbon materials have poor capacitance because of their formless morphology, hindered aggregation, and ineffective electrical double-layer capacitive reactions. Among various transition metal oxides, titanium oxides are considered ideal electrode materials for charge storage due to their stable structures and appropriate operating potential. Numerous studies have shown that titanium oxides with a layer structure and linked framework might be advantageous for rapidly inserting aqueous ions by providing favorable transport and distinctive pseudocapacitance [14,15].

Among numerous transition metal oxides, titanium dioxide (TiO_2_) is one of the most extensively employed semiconductors and photocatalysts in dye-sensitized solar cells [16]. High surface-to-volume ratio, abundant availability, good conductivity, high energy density, and outstanding light absorption properties make TiO_2_ a valuable material for SC electrode [17]. In particular, nanostructured TiO_2_ is increasingly garnering attention as an electrode material in SCs because of its large specific surface area and low cost compared with other metal oxides. For instance, Lee et al. [18] presented mesoporous MnO_2_ microparticles mixed with TiO_2_ nanoparticles, and the TiO_2_/MnO_2_ composite electrode delivered a specific capacitance of 279 F/g at 10 mV/s with 73.4% capacitance retention after 5000 cycles. However, TiO_2_’s significant limitations, such as its limited electrical conductivity and sluggish ion diffusion, restrict its effectiveness [19]. Therefore, to fabricate low-cost and practically applicable SCs based on TiO_2_, strategies that have the potential to improve the performance of TiO_2_ are in great demand. Alternatively, TiO_2_’s good chemical stability and eco-friendliness make it a promising material for the synthesis of composite electrodes in numerous charge storage devices.

Since polymer membranes offer considerable electrochemical stability and are simple to produce, such membranes have been used in SCs. To improve the membrane’s physical, electrical, and mechanical characteristics, polymer composite membrane preparation is a technology added to boost conductivity at ambient temperature [20]. Among several polymers, polyvinylidene fluoride (PVDF) is an exceptionally non-reactive thermoplastic fluoropolymer synthesized via the polymerization of vinylidene difluoride. It has a good resistance against the range of solvents (including basic and acidic ones) and, compared to other fluoropolymers, possesses a low density [21].

Therefore, considering the widespread application of TiO_2_ nanoplates due to their excellent characteristics that lead to enhanced chemical and thermal stability. In this work, the preparation of TiO_2_ nanoplates/PVDF membrane (denoted as TiO_2_/PVDF) was reported using a straightforward hydrothermal process. Because of its membrane-like structure, the composite material has supported the development of an effective electrode for SC applications. Experimental evidence exhibit that the TiO_2_/PVDF electrode has the advantageous characteristics of a high specific surface area and rapid charge transfer. Thus, the TiO_2_/PVDF electrode showed a maximum specific capacitance of 283.74 F/g at a current density of 1 A/g. In addition, the TiO_2_/PVDF electrode retained 91% of its capacitance after 8000 cycles, indicating its viability for use in the emerging field of energy storage.

## 2. Results and Discussion

**Figure 1:**
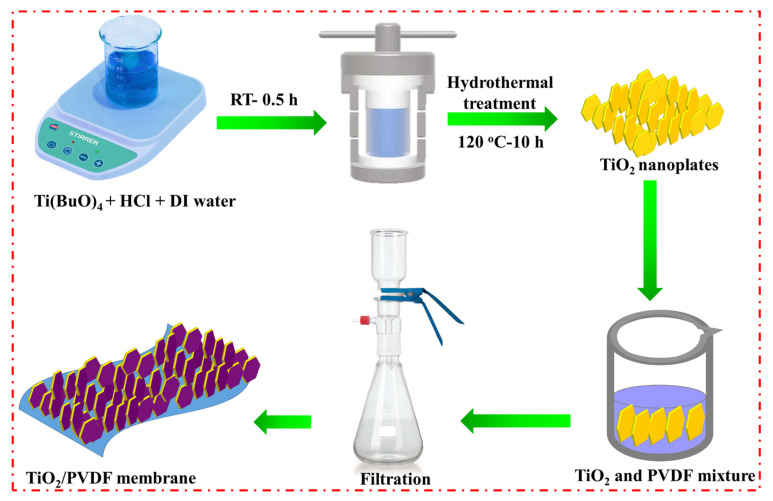
Schematic diagram of the synthesis strategy of TiO_2_/PVDF membrane.

Figure 1 provides a schematic illustration of the synthesis procedure for TiO_2_/PVDF membranes using a straightforward and facile hydrothermal strategy. During the fabrication method, titanium tetra-n-butoxide was converted into TiO_2_ nanoplates, which are then used to embellish the TiO_2_/PVDF membrane. As a result, a TiO_2_/PVDF membrane was fabricated with the ease of a simple vacuum filtration process.

**Figure 2:**
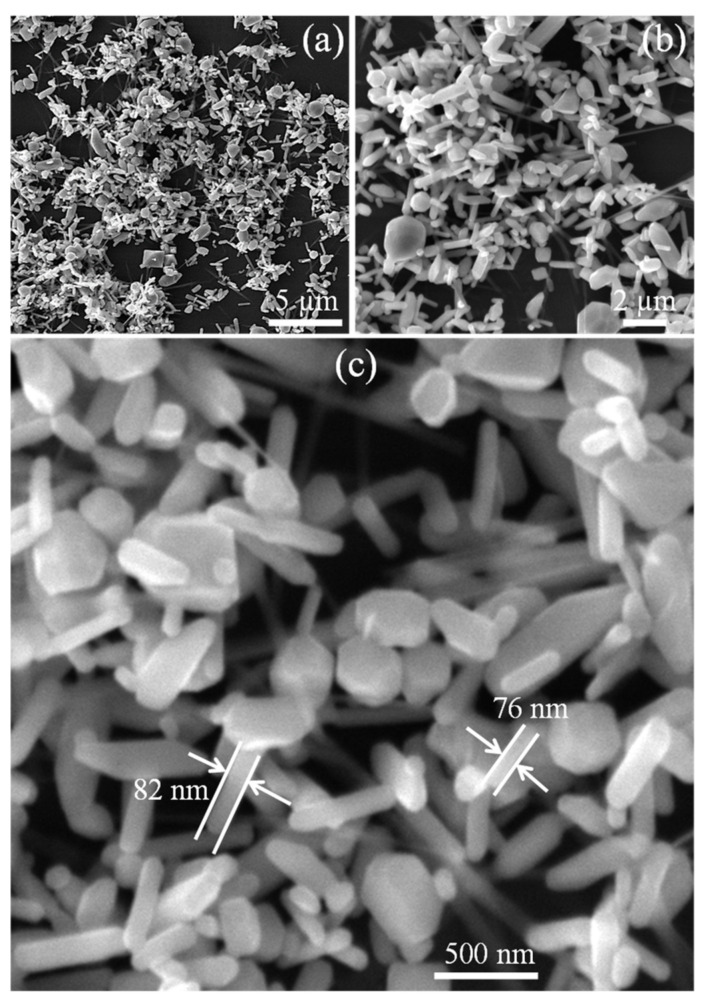
SEM images of TiO_2_ at; (**a**,**b**) Low-magnification; (**c**) High-magnification.

FE-SEM was used to investigate the structure and morphology of the as-synthesized TiO_2_ product. Low and high magnification SEM images (Figure 2a–c) exhibit the homogenous dispersion of TiO_2_ nanoplates with an average size of 79 nm, demonstrating the outstanding TiO_2_ nanoplates adhesion and effective grafting on PVDF membrane. Simultaneously, particle size reduction substantially enhanced the specific surface area of TiO_2_ and the number of its active sites in solution [22]. Uniform dispersion of these TiO_2_ nanoparticles was necessary to maximize the contact area between TiO_2_ nanoparticles and PVDF. As a result, the TiO_2_/PVDF membrane with tiny and evenly dispersed TiO_2_ nanoparticles was expected to have a wide range of applications in the field of electrochemical energy storage. It should be noted that the morphology of the TiO_2_/PVDF remained almost same of like TiO_2_ due to the small amount of the PVDF. These TiO_2_ nanoplates combined to form a unique porosity network which established wide channels for the fast transport of K^+^ and OH^−^ ions. Consequently, electrolyte ions may readily interact with the surface of most nanoplates. This decreased the ion diffusion distance, which ultimately resulted in an improvement in the electrochemical performance.

**Figure 3:**
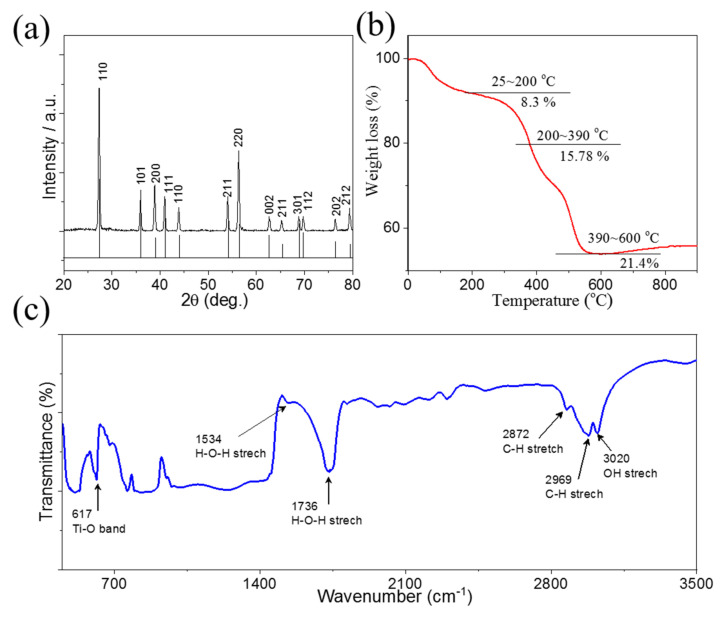
(**a**) XRD patterns of TiO_2_; (**b**) TGA curve of TiO_2_; (**c**) FTIR spectra.

Figure 3a displays the X-ray diffraction (XRD) patterns of the as-synthesized TiO_2_. All diffraction peaks were strong, indicating that the sample had high crystallinity. The XRD analysis showed that the TiO_2_ pattern was accurately indexed with the standard tetragonal rutile phase (JCPDS Card No. 21-1276) [23]. The thermogravimetric analysis (TGA) was used to study the thermal stability of TiO_2_ from 0 to 1000 °C in an air environment at a ramping rate of 5 °C/min. Figure 3b exhibits that the TiO_2_ nanoplates lost just 8.3% of their weight because of the absorbed water process when subjected to temperatures between 25 and 200 °C. TiO_2_ nanoplates showed a weight loss of 15.78% when subjected to temperatures between 200 and 390 °C. The reduction of -OH groups on the surface of the TiO_2_ nanoplates was primarily responsible for this result. Furthermore, the weight of the TiO_2_ nanoplates decreased by 21.4% when heated to temperatures ranging from 390 to 600 °C [24]. The buoyancy effect of TGA equipment may account for the modest gain in weight seen [25,26]. Finally, stability up to 800 °C demonstrated the suitability of TiO_2_ for high-temperature applications, where conventional materials would degrade. Fourier transformed infrared (FTIR) was also performed to verify the composition and bonding structure of TiO_2_. Figure 3c shows that TiO_2_ had a strong band at 670 cm^−1^, which was associated with the stretching vibration of Ti–O [27], and the peaks at 1534 and 1736 cm^−1^ can be related to the stretching vibration of H-O-H. Additionally, the peaks located at 2872 and 2969 cm^−1^ may be attributed to the C-H stretching vibration [28], and a peak at 3020 cm^−1^ can be associated with O-H stretching vibration, indicating that the utilization of TiO_2_ might increase the hydrophilicity of the membrane [29].

**Figure 4:**
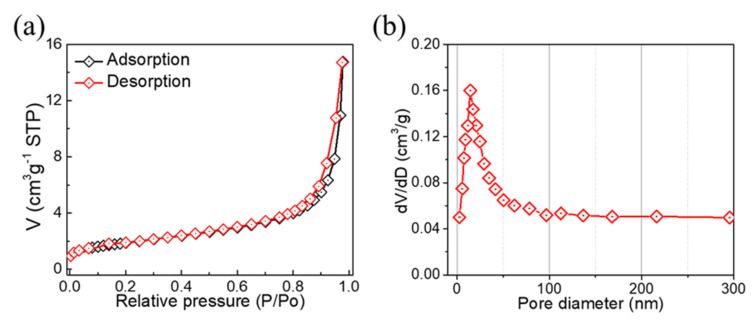
(**a**) N_2_ adsorption/desorption isotherm; (**b**) Typical pore size distribution of TiO_2_.

The N_2_ adsorption/desorption isotherms of TiO_2_ are shown in Figure 4a, which exhibit a hysteresis loop at P/P_o_ > 0.7 and confirm a typical type IV adsorption behavior, indicating that TiO_2_ likely possesses a mesoporous structure. Furthermore, when the relative pressure P/P_o_ > 0.8, the adsorption/desorption isotherm was steep because, during cohesion and evaporation, the relative pressure was centered, suggesting that the TiO_2_ exhibited mesoporous characteristics. The BET-specific surface area of TiO_2_ was calculated to be 17.613 m^2^/g [28].

Figure 4b depicts the BJH pore size distribution curve, and an average pore size of 23.4 nm was observed, indicating the presence of sizable pores. Because of the large pore size and increased specific surface area, there would be more electroactive sites and easier access for the electrolyte ions. This would improve the electrode materials’ electrochemical performance for storing charge [30].

**Figure 5:**
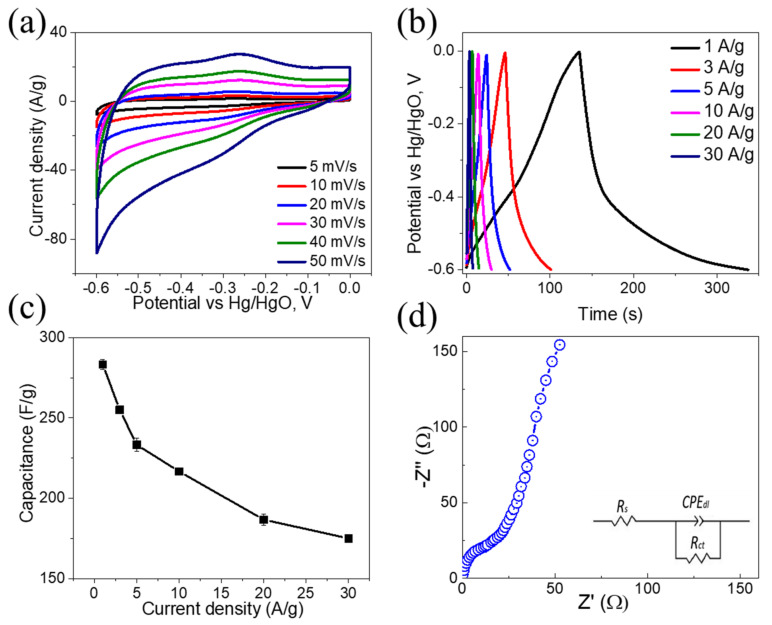
Electrochemical measurements of a TiO_2_/PVDF membrane electrode in the aqueous electrolyte; (**a**) CV profiles at different scan rates; (**b**) GCD profiles at different current densities; (**c**) Specific capacitance versus current density; (**d**) Nyquist plot.

The electrochemical performance of the TiO_2_/PVDF membrane for the energy storage device as a supercapacitor electrode was investigated in a three-electrode setup, as shown in Figure 5. The CV profiles of the TiO_2_/PVDF electrode were measured in the potential window of −0.6 to 0.0 V and at various scan rates ranging from 5 to 50 mV/s, and the results are presented in Figure 5a. The nearly-rectangular CV profiles of the TiO_2_/PVDF electrode suggested that the electrode material exhibits exceptional capacitive performance and high-rate capability. The CV profiles possess remarkable stability, even at high scan rates, demonstrating the low polarization resistance of the TiO_2_/PVDF electrode [31]. In the CV profiles, the robust current response is another demonstration of the outstanding charge storage capacity and quick electrolyte ion accessibility. When the scan rate was low, both the inner and outer surfaces of the sample could be utilized as charge storage. However, when scan rates were higher, the diffusion of OH^−^ ions was more likely to occur exclusively on the surface, and only a negligible fraction of OH^−^ ions could insert into the inner surface. This phenomenon demonstrates that the porous characteristics of the TiO_2_/PVDF membrane allow it to accommodate the OH^−^ ions [32,33,34].

Figure 5b exhibits the GCD profiles of TiO_2_/PVDF electrode at different current densities from 1 and 30 A/g, where symmetric GCD profiles are attributed to the pseudocapacitive characteristics of the electrode. Also, each GCD profile shows great symmetry without a discernible plateau, indicating high Coulombic efficiency and good reversibility. Figure 5c depicts the specific capacitance (C_sp_ (F/g)) of the TiO_2_/PVDF electrode (according to Equation (1)) from the GCD profiles at current densities ranging from 1 to 30 A/g. The TiO_2_/PVDF electrode delivered a C_sp_ of as high as 283.74, 255.09, 233.41, 216.65, 190.18, and 174.94 F/g at 1, 3, 5, 10, 20, and 30 A/g, respectively. After increasing the current density from 1 to 30 A/g, the TiO_2_/PVDF electrode showed a capacitance retention of 61.6%, which demonstrated a remarkable rate capability at high current density. The C_sp_ of the electrode remained as a maximum of 174.94 F/g even though the current density was increased to 30 A/g (capacitance retention of ~61.6%). An EIS test was also performed to understand the impedance of the TiO_2_/PVDF electrode, and the Nyquist plot is shown in Figure 5d. The equivalent series resistance (R_s_) of the electrode is characterized in the high-frequency zone by the intercept at the real axis, which was ~0.84 Ω (includes the internal resistance of electrode material and electrolyte resistance). The small value of R_s_ implies that there is excellent electrical conductivity between the electrode material and the current collector. The radius of a semi-circle corresponds to charge-transfer resistance (R_ct_), which is ~0.99 Ω. In the low-frequency zone, the quasi-vertical line represents the Warburg impedance Z_w_ of the electrolyte diffusion. The slope of the line is associated with the ionic diffusion of the electrolyte to the electrode surface. The EIS curve was fit using an equivalent electrical model created with the help of ZSimpWin. This model included the R_ct_, R_s_, and constant phase element (CPE). These values can be seen in the inset of Figure 5d [35,36,37]. It seems that TiO_2_/PVDF electrode with small R_s_ and R_ct_, became the most electrochemically active compared to MoS_2_ (R_s_ ~4 Ω and R_ct_ ~4.3 Ω) [35], b-TiO_2_ (R_s_ ~1.60 Ω and R_ct_ ~15.3 Ω) [38] and ICS-TiO_2_ (R_s_ ~7.5 Ω and R_ct_ ~11.3 Ω) [39] electrodes.

**Figure 6:**
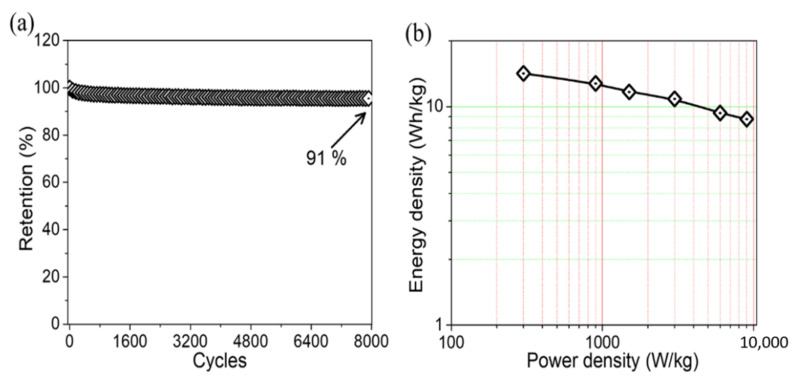
(**a**) Cycling performance of the TiO_2_/PVDF electrode; (**b**) Energy density versus power density.

The cycling stability analysis is an essential parameter for SC electrode materials. Figure 6a shows the cycling stability test of the TiO_2_/PVDF electrode, which was carried out by continuously operating it for 8000 GCD cycles at a current density of 10 A/g. The TiO_2_/PVDF electrode retained a capacitance of 91% after 8000 cycles, demonstrating the extremely high stability and excellent energy storage capabilities of the TiO_2_/PVDF electrode throughout GCD cycles. This enhanced electrochemical performance of the synthesized TiO_2_/PVDF electrode in an aqueous electrolyte may be due to the membrane-like morphology of the TiO_2_/PVDF electrode and its increased electrical conductivity [40]. The importance of the TiO_2_/PVDF electrode can be better understood by comparing its electrochemical performance with that of different composites.

Table 1 shows the overall performance of the TiO_2_/PVDF electrode compared to that of previously published related electrodes in terms of specific capacitance and cycling stability, which shows the superior performance of the TiO_2_/PVDF electrode. To analyze the overall performance of SC, the power density vs. energy density (Ragone plots) for the TiO_2_/PVDF electrode was calculated according to Equations (2) and (3) and displayed in Figure 6b. The estimated E and P of the TiO_2_/PVDF electrode were 14.78, 12.76, 11.68, 10.84, 9.34, and 8.76 Wh/kg and 300.24, 900.72, 1501.2, 3002.4, 6004.8, and 9007.2 W/kg, respectively. Since E is directly dependent on specific capacitance, a higher specific capacitance in the TiO_2_/PVDF electrode led to a higher E. A maximum energy density of 14.18 Wh/kg was achieved while maintaining a power density of 300.24 W/kg. The experimental data demonstrates that TiO_2_/PVDF membrane was a viable electrode material for SCs.

## 3. Experimental Section

### 3.1. Synthesis of TiO_2_ Nanoplates

To fabricate TiO_2_ nanoplates, a hydrothermal process was applied to titanium (IV) butoxide (Ti(BuO)_4_) (Sigma-Aldrich), hydrochloric acid (HCl), ethanol, and deionized (DI) water. After adding Ti(BuO)_4_ (4.8 g) to 15 mL of HCl, 12 mL of DI water was added. The resultant complex was agitated using a magnetic stirrer at room temperature (RT) for 30 min. The solution was then placed in an autoclave and heated in an oven at a temperature of 120 °C for reaction durations of 10 h. Then, the autoclave was left to cool at an ambient temperature naturally, and TiO_2_ nanoplates were collected and rinsed with DI water.

### 3.2. Synthesis of TiO_2_/PVDF Membrane

For the typical synthesis of TiO_2_/PVDF membrane, a solution was prepared by dissolving PVDF in dimethylformamide (DMF) (containing PVDF powder of 0.1 g dissolved in 10 mL of DMF) and stirring it to completely dissolved PVDF. Then, 0.9 g of TiO_2_ nanoplates were mixed in a PVDF/DMF solution. A hand-casting knife with a 150 µm gap was used to mix the slurry uniformly. Afterwards, the mixture was vacuum filtered through a mesh (0.45 µm), and the resulting TiO_2_/PVDF membrane was collected on the filter paper. Finally, the TiO_2_/PVDF membrane was washed with DI water to eliminate any residuals of the solvent and dried at 60 °C.

### 3.3. Physical Characterization of TiO_2_/PVDF Membrane

To investigate the morphology of TiO_2_, a field emission scanning electron microscope (FESEM, HITACHI SU8220, Hitachi High-Technologies, Tokyo, Japan) was used. The crystal structure was analyzed via an X-ray diffractometer (XRD, Philips X’Pert Pro Analytical, Philips, Singapore) using monochromatic radiation (Cu-Kα; = 0.15406 nm). The thermogravimetric analysis (TGA) measurement was carried out in an air environment at a 5 °C/min heating rate from room temperature to 1000 °C. Fourier transformed infrared (FTIR) spectra (Vector-22 spectrometer, Nicolet IS 10, Thermo Scientific, Waltham, MA, USA) was used. For the analysis of the surface area and pore size, Brunauer-Emmett-Teller (BET) and Barret-Joyner-Halenda (BJH) analytical curves with the (Micromeritics ASAP2460, Micromeritics, Shanghai, China) analyzer were acquired, respectively.

### 3.4. Electrochemical Characterization of TiO_2_/PVDF Membrane

The electrochemical measurements were carried out with a three-electrode configuration using an electrochemical workstation (CHI 660E, Corrtest Instruments, Wuhan, China). The as-synthesized TiO_2_/PVDF (1.5 × 2.5 cm^2^) membrane with a mass loading of 1.1 mg/cm^2^ was used as a working electrode for Hg/HgO, and platinum wire was used as a reference and counter electrodes, respectively, in an aqueous electrolyte containing 6 M KOH. A cyclic voltammogram (CV) was performed over a potential window range of −0.6 to 0.0 V. Galvanostatic charge-discharge (GCD) was carried out over the same potential window range (−0.6 to 0.0 V) at varying current densities (1 to 30 A/g). The electrochemical impedance spectroscopy (EIS) was performed at frequencies ranging from 0.001 to 100 kHz at open circuit conditions with a 5-mV amplitude.

The following Equation (1) was used to determine the specific capacitance (C_sp_ (F/g)) of the TiO_2_/PVDF membrane:

(1) $$\mathrm{Csp}=\frac{1}{mv\left(V_{f}-V_{i}\right)}\;\int_{V_{i}}^{V_{f}}I\;dV$$

where *m*(g) is the mass of active material, *v*(V/s) is the scan rate, *I*(A) is the applied current, and Δ*V*(*V_f_* − *V_i_*) is the potential window.

The following Equations (2) and (3) were used to calculate the energy density E (Wh/kg) and the power density P (kW/kg). Where C_sp_ is the electrode’s specific capacitance and Δ*V* is the potential, t_d_ is the discharge time.

(2) $$E=0.139\;C_{sp}∆V^{2}\;$$

(3) $$P=\frac{E}{t_{d}}\;$$

## 4. Conclusions

In summary, we have demonstrated TiO_2_/PVDF membranes fabricated via a cost-effective and straightforward hydrothermal strategy and employed as electrode material for energy storage applications. The electrochemical investigations of the as-prepared TiO_2_/PVDF electrode using a three-electrode configuration in an aqueous electrolyte exhibited exceptional performance and delivered a specific capacitance of 283.74 F/g at 1 A/g. The TiO_2_/PVDF electrode retained a capacitance of 91% after 8000 cycles. Additionally, a maximum energy density of 14.18 Wh/kg of TiO_2_/PVDF electrode was achieved while maintaining a power density of 300.24 W/kg. Our findings demonstrate that the TiO_2_/PVDF electrode has the potential to deliver an adjustable electrochemical performance, which is a prerequisite for its commercialization and use in the development of state-of-the-art supercapacitors.

## Figures and Tables

**Table 1:** A comparison table of the electrochemical performance of previously published works with the as-prepared TiO_2_/PVDF membrane for supercapacitor applications.

Sr. No.	Electrode Material	Electrolyte	Specific Capacitance (GCD/CV)	No. of Cycles (n)	Capacitance Retention (%)	Ref.
1	TiO_2_/PVDF membrane	KOH	283.74 F/g (1 A/g)	8000	91	This work
2	CB)/TiO_2_	H_2_SO_4_	236 F/g (10 mV/s)	10,000	90.3	[41]
3	Fe-TiO_2_/C nanofibers	KOH	137 F/g (5 mV/s)	-	-	[42]
4	TiO_2_-graphene composites	KOH	84 F/g (10 mV/s)	1000	87.5	[43]
5	N-TiO_2_/NG	Na_2_SO_4_	205.1 F/g (1 mV/s)	5000	78.8	[44]
6	BC-G-TiO_2_	H_2_SO_4_	250.8 F/g (2 A/g)	100	84.4	[45]
7	TiO_2_-activated carbon	Na_2_SO_4_	92 F/g (5 mV/s)	5000	89.9	[46]
8	TiO_2_-CNT	H_2_SO_4_	110 F/g (0.05 mA/cm^2^)	-	-	[47]
9	rGO/TiO_2_ NR/rGO	Na_2_SO_4_	114.5 F/g (5 mV/s)	4000	85	[48]
10	TiO_2_-carbon NFs	KOH	106.57 F/g (1A/g)	2000	84	[16]

## Data Availability

Not applicable.
